# Effect of the defect localization and size on the success of third-generation autologous chondrocyte implantation in the knee joint

**DOI:** 10.1007/s00264-020-04884-4

**Published:** 2020-12-06

**Authors:** Thomas R. Niethammer, David Gallik, Y. Chevalier, Martin Holzgruber, Andrea Baur-Melnyk, Peter E. Müller, Matthias F. Pietschmann

**Affiliations:** 1 Department of Orthopaedic Surgery, Physical Medicine and Rehabilitation, University Hospital of Munich (LMU), Campus Grosshadern, Marchioninistr. 15, 81377 Munich, Germany; 2 Institute of Clinical Radiology, Ludwig-Maximilians-University Munich, Grosshadern Campus, Marchioninistr. 15, 81377 Munich, Germany

**Keywords:** ACI, Autologous chondrocyte implantation, Defect size, Cartilage defect, Localization

## Abstract

**Introduction:**

Femoral and patellar cartilage defects with a defect size > 2.5 cm^2^ are a potential indication for an autologous chondrocyte implantation (ACI). However, the influence of the localization and the absolute and relative defect size on the clinical outcome has not yet been determined. The purpose of this study is to analyze the influence of the localization and the absolute and relative defect size on the clinical outcome after third-generation autologous chondrocyte implantation.

**Methods:**

A total of 50 patients with cartilage defects of the knee were treated with third-generation autologous chondrocyte implantation (Novocart® 3D). A match paired analysis was performed of 25 treated femoral and 25 treated patella defects with a follow-up of three years. MRI data was used to do the manual segmentation of the cartilage layer throughout the knee joint. The defect size was determined by taking the defect size measured in the MRI in relation to the whole cartilage area. The clinical outcome was measured by the IKDC score and VAS pre-operatively and after six, 12, 24, and 36 months post-operatively.

**Results:**

IKDC and VAS scores showed a significant improvement from the baseline in both groups. Femoral cartilage defects showed significantly superior clinical results in the analyzed scores compared to patellar defects. The femoral group improved IKDC from 33.9 (SD 18.1) pre-operatively to 71.5 (SD 17.4) after three years and the VAS from 6.9 (SD 2.9) pre-operatively to 2.4 (SD 2.5) after three years. In the patellar group, IKDC improved from 36.1 (SD 12.6) pre-operatively to 54.7 (SD 20.3) after three years and the VAS improved from 6.7 (SD 2.8) pre-operatively to 3.4 (SD 2.) after three years. Regarding the defect size, results showed that the same absolute defect size at med FC (4.8, range 2–15) and patella (4.6, range 2–12) has a significantly different share of the total cartilaginous size of the joint compartment (med FC: 6.7, range 1.2–13.9; pat: 18.9, range 4.0–47.0). However, there was no significant influence of the relative defect size on the clinical outcome in either patellar or femoral localization.

**Conclusion:**

Third-generation autologous chondrocyte implantation in ACI-treated femoral cartilage defects leads to a superior clinical outcome in a follow-up of three years compared with patellar defects. No significant influence of the defect size was found in either femoral or patellar cartilage defects.

## Introduction

Full-thickness cartilage defects are pre-arthritic lesions and can produce significant pain and disability for patients [1, 2]. The intrinsic regeneration capability of the cartilage is very limited and the healing likelihood of once damaged cartilage is reduced. Many studies have proven that autologous chondrocyte implantation (ACI) represents an appropriate method for treatment of larger full-thickness cartilage defects in knees and leads to significant improvement [3–9].

Since the first-generation ACI, using a periosteal flap, there have been many improvements in this procedure including using a collagenous membrane (second-generation ACI) or a 3D collagenous scaffold (third generation) [10]. The available studies researching the possible influencing factors (site, lesion size, etiology, age, location etc.) on the outcome of ACI have shown various results [11–18]. As for the localization, the defects on femoral condyle reached excellent mid-term and long-term clinical outcomes, while patellar-located lesions were associated with less successful clinical outcomes [11–13, 19]. The improvement of the ACI technique and the concomitant correction of the patellofemoral malalignment improved the outcome of the patellar defects [15, 17]. Despite these improvements, using the ACI on the patella remains controversial and with unclear results, which are worth researching.

There are only a few studies analyzing the third generation of the ACI focusing on the localization of the defect and comparing the femoral with patellar defects [20, 21]. According to current recommendations, the defect size of 2.5–3 cm^2^ and larger is regarded as a potential indication for the ACI [7, 22]. However, it is not known whether this “critical” limit is identical for the different knee compartments as patella and condyles or whether a differentiated size consideration with relative defect size (damaged area in relation to the total compartment) for these compartments is needed. The correlation between the outcome and the absolute defect size measured intra-operatively was the focus of previous studies showing various results [23, 24]. The relative defect size had not been researched and evaluated until this study.

The aim of our research is to analyze the influence of the localization and the defect size on the clinical outcome following third-generation ACI. Our hypothesis was that patellar defects and defects with a bigger share on the whole cartilage layer of the knee compartment lead to less successful outcomes.

## Methods

Our data were captured between 2004 and 2018 with a local Institutional Review Board approval. All patients from our database with femoral and patellar cartilage defects of the knee classified as International Cartilage Repair Society (ICRS) grades III and IV were included in our prospective study and treated with third-generation ACI with intact meniscus and corresponding joint surfaces. We intended to perform a matched pair analysis. The criteria for the pair matching were age, sex, body mass index, numbers of treated defects, and the intra-operatively measured absolute defect size (Table 1). The first group represented 25 patients with medial condyle defects, while the second group consisted of 25 matched patients with patellar defects. All patients were treated with Novocart® 3D (TETEC AG, Reutlingen, Germany), a third-generation ACI.

**Table 1:** Patient characteristics

		Med FC	Patella
Number of patients		25	25
Gender, *n* (%)	Male	11 (44)	10 (40)
	Female	14 (56)	15 (60)
Intraoperative defect size in cm^2^ (range; SD)		4.83 (2–15; 2.79)	4.61 (2–12; 2.39)
BMI in kg/m^2^ (range; SD)		27.3 (20–36; 4.68)	26.3 (19–35; 4.83)
Age in years (range; SD)		34.6 (15–53; 12.38)	33.2 (13–56; 12.51)

**Fig. 1:**
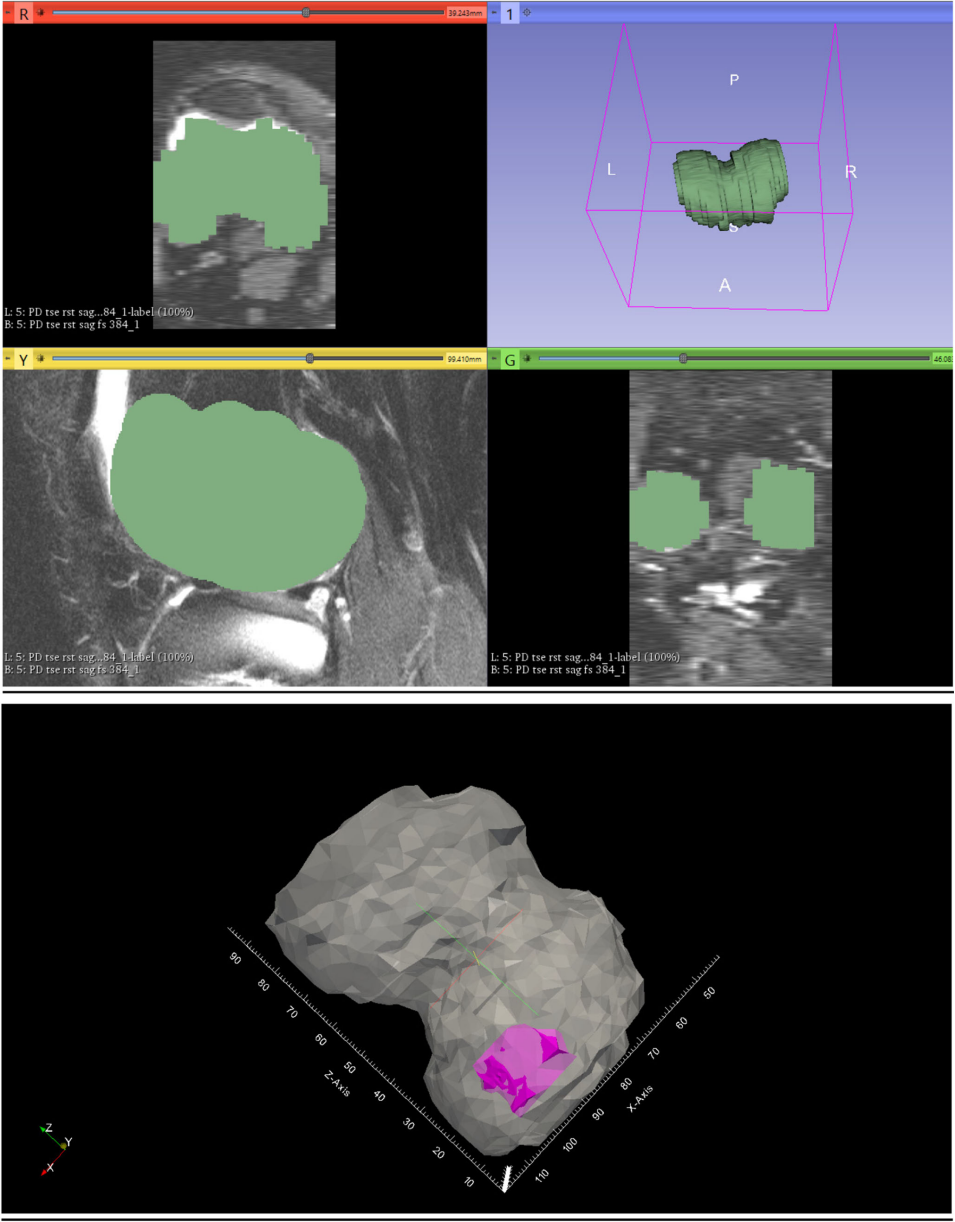
The DICOM (Digital Imaging and Communications in Medicine) datasets were used in the open source software 3D Slicer to do the manual segmentation of the cartilage layer and of the defect (**a**). In the further post-processing using the ParaView and YBones software, the share of the defect on the cartilage layer of the knee compartment, which represented the relative defect size in %, was calculated. The violet part in the picture is the femoral defect, and the white part represents the cartilage layer (**b**)

For precise detection of the absolute defect size, magnetic resonance imaging (MRI) examinations were performed (Magnetom-Sonata; Siemens, Germany) with a conventional circular polarizing 1-channel knee coil three months post-operatively. The following sequences were performed: the fast spin-echo (dual T2-FSE), the fat-saturated gradient echo (3D FS GE), the proton-weighted fat-saturated T1-weighted sequence, and a fast-low-angle shot sequence (FLASH) with selective water excitation, all sequences suitable for the measurement of articular cartilage in the knee joint. DICOM (Digital Imaging and Communications in Medicine) data sets were used in the open source software 3D Slicer to do a manual segmentation of the cartilage layer and of the defect. 3D Slicer is a free software platform for the analysis and visualization of medical images. After the segmentation of the cartilage layer and the cartilage defect, we determined the relative defect size by putting the defect size into relation with the cartilage volume size of the medial femoral condyle/patella, which represented the relative defect size in % (Fig. 1) using the ParaView (Kitware, Clifton Park, New York, USA) and custom-made software [25].

Patient-reported outcomes were measured pre-operatively and at six, 12, 24, and 36 months post-operatively using the clinical scores IKDC (International Knee Documentation Committee) subjective knee form and a visual analog scale (VAS). Additionally, patient-specific parameters such as age, gender, and body mass index (BMI), as well as defect-specific data such as defect size or localization, were collected.

For the statistical analysis of the clinical data, the statistic program SPSS (Statistical Package for the Social Sciences, Chicago, IL, USA) was used. For the detection of significant differences between the two groups at the same time of investigation, the Wilcoxon or Friedman test was carried out for paired samples. To compare multiple groups of non-related samples at one point, the Mann–Whitney *U* test was used. Pearson’s correlation models were used to distinguish associations between influencing factors and clinical knee scores. Our primary outcome parameter was the IKDC score, secondary outcome the VAS. A post hoc power analysis was performed with an effect size of 0.916. A statistically significant result of *p* < 0.05 was reported.

## Results

There were a total of 50 patients in two matched groups with 25 patients in each study group. As previously explained, all the known influencing factors were removed by matching. The average age of the femoral group was 34.6 years (15–53), 11 male and 14 females, with the mean body mass index (BMI) of 27.3 kg/m^2^ (20–36). The mean intra-operative defect size was 4.8 cm^2^ (2–15). In the matched pair group with ACI patients, in the patellar group, the average age was 33.3 years (13–56), ten male and 15 females with a mean BMI of 26.3 kg/m^2^ (19–35) and average intra-operative defect size of 4.6 cm^2^ (2–12). Patient characteristics are described in Table 1. In 6 (24%) cases in the femoral group, concomitant surgery was carried out (4 cancellous bone grafting and 2 anterior cruciate ligament reconstructions). In the patellar group, nine (36%) concomitant surgery (8 stabilization of the medial retinaculum and 1 high tibial osteotomy) were performed. Previous surgery was performed in nine cases in the patellar group (bone marrow stimulation *n* = 7, cartilage shaving *n* = 1, high tibial osteotomy *n* = 1) and nine cases in the femoral group (bone marrow stimulation *n* = 8, refixation flake fracture *n* = 1).

### Localizations

**Fig. 2:**
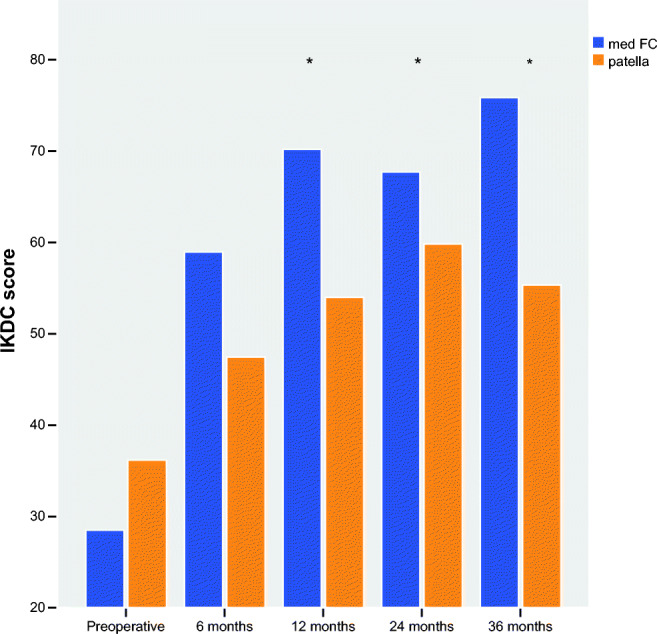
The evolution of the IKDC results of the two groups over a period of three years. In both groups, there was a significant improvement in comparison to pre-operative values at all timepoints. At 12, 24, and 36 months was the IKDC value of the med FC group significantly better (marked with *) than the patellar group (6 months *p* = 0.101, 1 year *p* = 0.0.016, 2 years *p* = 0.030, and after 3 years *p* = 0.009)

In both groups, a significant IKDC improvement within the group in comparison to pre-operative values (pre-operative vs. 6, 12, 24, 36 months; *p* < 0.05) (Fig. 2) was found at all timepoints. In the femoral group, the IKDC subjective score was 33.9 (3–67) pre-operatively and 56.4 (28–93) after six months. Afterwards, we observed further increasing IKDC score to 66.7 (25–95) after 12 months and 70.2 (48–100) after two years. The maximum value 71.5 (40–100) was reached after three years (Table 2). The patellar group showed an IKDC subjective score of 36.1 (14–65) pre-operatively. Six months pos-toperatively, it was 47.5 (22–78) and the 12-month value was 54.3 (23–81). After two years, the maximum IKDC value was reached with 57.7 (16–93). A slight decrease was seen after three years to 54.7 (22–92).

**Table 2:** Clinical outcomes of medial femoral condyle (med FC) and patella group over the time

		Pre-operative	6 months	12 months	24 months	36 months
Med FC	IKDC (range; SD)	33.9 (3–67; 18.13)	56.4 (28–93; 17.14)	66.7 (25–95; 19.35)	70.2 (48–100; 14.66)	71.5 (40–100; 17.47)
	VAS at movement (range; SD)	6.90 (0–10; 2.90)	3.60 (0–9; 3.10)	2.81 (0–9; 2.74)	1.83 (0–8; 2.23)	2.40 (0–9; 2.50)
	VAS at rest (range; SD)	2.63 (0–8; 2.69)	0.57 (0–6; 1.36)	0.87 (0–5; 1.44)	0.53 (0–4; 1.04)	0.78 (0–5; 1.48)
Patella	IKDC (range; SD)	36.1 (14–65; 12.59)	47.4 (22–78; 17.46)	54.3 (23–81; 15.48)	57.7 (16–93; 18.59)	54.7 (22–92; 20.31)
	VAS at movement (range; SD)	6.71 (0–10; 2.81)	4.71 (1–10; 2.63)	4.14 (1–9; 2.45)	4.51 (1–10; 3.15)	3.40 (0–9; 2.49)
	VAS at rest (range; SD)	2.66 (0–9; 3.06)	1.8 (0–5.5; 1.92)	0.57 (0–3.7; 0.95)	0.7 (0–3.4; 0.99)	1 (0–3; 1.1)

Comparing the IKDC results of our two groups over a period of three years, we found a significant difference between the two groups. The IKDC score showed a significant difference between the two groups one to three years post-operatively (*p* < 0.05). At all post-operative timepoints, the IKDC value of the femoral group was better than of the patellar group. Only after six months was the difference (*p* > 0.05) statistically not significant. In the following timepoints, significant differences of IKDC score were noticed (after 1 year *p* = 0.016, 2 years *p* = 0.030, and 3 years *p* = 0.009) (Fig. 1; Table 2).

**Fig. 3:**
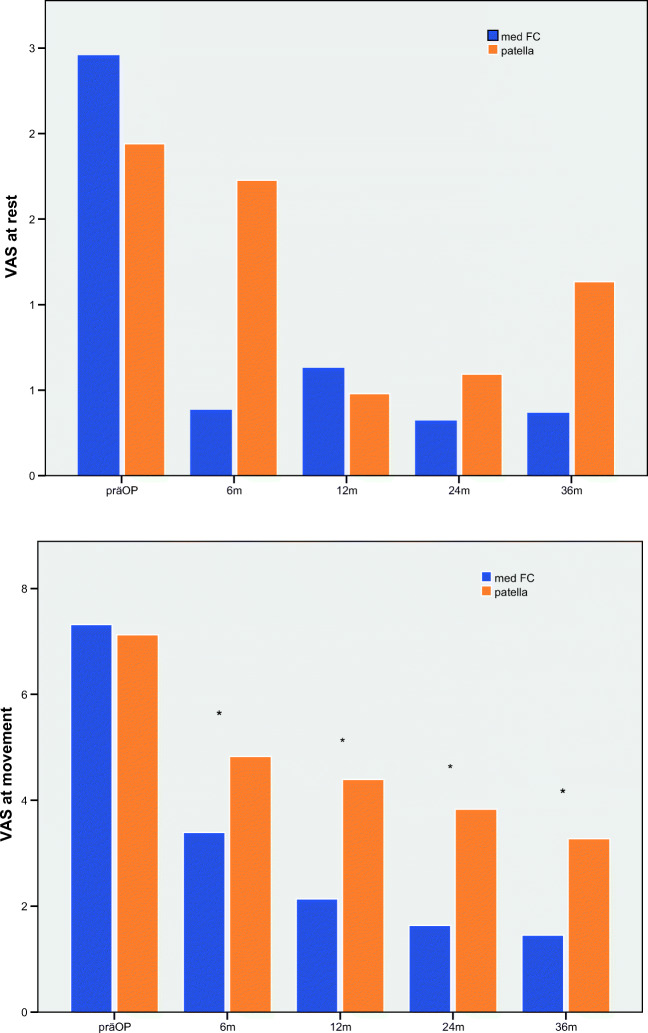
The comparison of the VAS at rest (**a**) and at movement (**b**) in med FC and pat group. The VAS at rest in med FC reached a significant improvement 6, 12, 24, and 36 months after ACI. In the pat group, a significant improvement (*p* < 0.05) of the VAS at rest scale was seen at post-operative measurements after 12 (*p* < 0.01) and 24 months (*p* < 0.002). As for the VAS at movement, there was a significant improvement measured at all times post-operatively in both medial femoral condyle (med FC) and patella groups (*p* < 0.02) (*Stands for significance in both group)

The results of the visual analog scale (VAS) also showed a statistically significant improvement over time. In the femoral group, the patients assessed the VAS at rest pre-operatively with an average of 2.6 (0–8). Post-operatively, the VAS at rest was 0.57 (0–6), 0.87 (0–5), 0.53 (0–4), and 0.78 (0–5) measured 6, 12, 24, and 36 months after ACI, which means a significant improvement at all timepoints (Table 2; Fig. 2). In the patellar group, a significant improvement (*p* < 0.05) of the VAS at rest scale was seen at post-operative measurements after 12 (*p* < 0.01) and 24 months (*p* < 0.002). As for the VAS at movement, there was a significant improvement measured at all times post-operatively in both femoral and patellar groups (*p* < 0.02). The rate of the VAS at movement in the femoral group pre-operatively was 6.9 (0–10) and improved to 1.8 (0–8) at 24 months and to 2.4 (0–9) at 36 months post-operatively. Similarly, in the patellar group, VAS at movement improved from 6.7(0–10) pre-operatively to 3.4 (0–9) after 36 months (Table 2; Fig. 3). Comparing the two groups, a statistically significant difference between the two groups was shown two years post-operatively for VAS at movement (*p* = 0.004) and six months post-op for VAS at rest (*p* = 0.005) (Table 2).

### Defect size

**Fig. 4:**
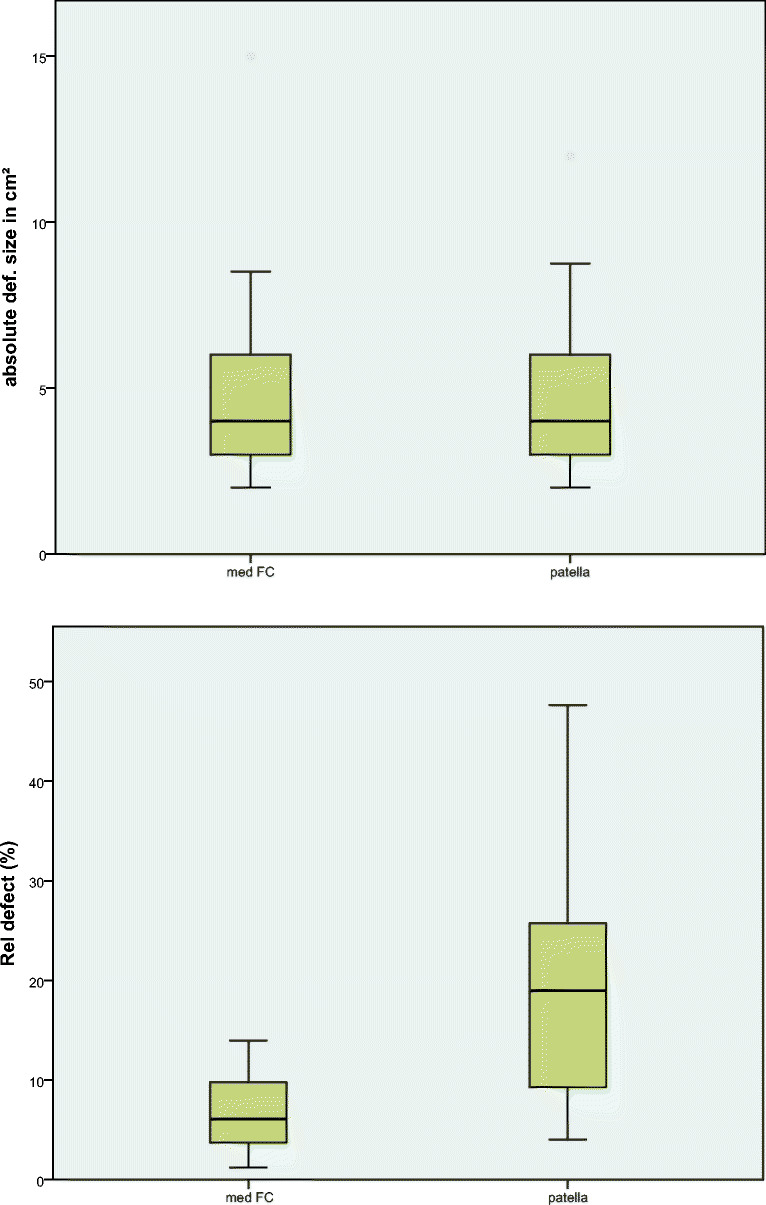
The same absolute defect size (**a**) at medial femoral condyle (med FC) and patella has a significantly different (*p* < 0.05) share on the total size of the joint compartment (relative defect size (**b**))

The mean relative defect share on the knee was 12.8% (1.2–47.7). In the femoral group, the average relative defect was 6.7% (1.2–13.9), while in the patellar group, it was 18.9% (4.0–47.7) (Fig. 4). Comparing these relative defects sizes, we found a significant difference between the medial femoral condyle and the patella group. This means that the same absolute defect size at medial femoral condyle and patella has a significantly different (*p* < 0.05) share on the total cartilage size of the joint compartment (relative defect size). It is graphically summarized in Fig. 4. In analyzing the influence of the relative defect size on the clinical outcome measured by IKDC and VAS, no statistically significant correlation was found (*p* > 0.05). As for the absolute defect size, no statistically significant correlation between the outcome and the absolute defect size was found.

## Discussion

The major findings of this study are that the third-generation ACI in femoral treated cartilage defects leads to a superior clinical outcome in a follow-up of three years compared with ACI-treated patellar cartilage defects and that the relative and absolute defect sizes do not have a significant influence on the outcome.

The third-generation ACI is an established and accepted method for the treatment of full-thickness cartilage defects in the knee as has been proven in many studies [6, 26]. The evidence on the success of the ACI has increased significantly during the past years [27–29]. The efficacy of this procedure has been demonstrated in multiple studies showing a positive effect, with increased function and pain reduction [3–5, 16, 19, 30]. The ACI with its cell expansion in vitro and the whole process is an expensive method of the cartilage therapy [31]. Therefore, the indication for using this procedure should be as clear as possible. All influencing factors are still the focus of many studies.

Since the introduction of ACI, several studies have described factors that influence its clinical outcome. There are patient-related (sex, age, BMI, physical activity) and defect-related (lesion size, location, prior procedures, etiology) aspects that can influence the results of the ACI [32]. The studies investigating the significant correlation of these factors with the outcomes have showed heterogeneous results. Some previous research described that female sex and chronic aetiology are associated with inferior outcomes [18, 33]. Regarding age and BMI as factors, some studies have shown the disadvantageous effects of higher patient age and BMI on the outcome of ACI [33–35].

Filardo et al. in a large matrix-associated ACI cohort presented the disadvantage of higher age with the degenerative aetiology and the benefit of male sex with higher physical activity. On the other hand, Vasiliadis et al. and Kon et al. described no association between clinical outcomes and age, and Gobbi et al. showed no correlation with aetiology [27, 33, 36]. Regarding prior procedures, a study regarding the negative influence of prior cartilage procedures has been published [37, 38].

In terms of influencing factors, in our study, we focused on the localization and the defect size. We removed other influencing factors by matching. The patellar-located lesions have been associated with lower clinical outcomes [19, 35], despite the correction of the patellar malalignment showed clinical improvement [17, 39, 40]. In our study, we only analyzed the third-generation ACI. There are few studies focusing on localization analyzing only the third-generation ACI to compare our results with. Gigante et al. did not see a significant difference between various localizations of the defect in his small sample of patients [41]. Filardo et al. compared third-generation ACI in patellar vs. trochlear region, with significant superiority for trochlear lesions. In another study from this group, condyle and trochlear lesions were compared with slightly better results for trochlear [42]. In the study of Gobi et al., ACI patients with trochlear lesions showed better results than those with patellar lesions [43]. We did not analyze the trochlear lesion at all. Meyerkort et al. described good clinical improvement with KOOS score > 70 five years after patellofemoral ACI but did not analyze the femoral localization [40].

Welsch et al. investigated the MACI comparing patella vs. med FC lesions and found no significant difference from the radiological point of view without clinical outcome scores [21]. Kon et al. analyzed the long-term results of the patellar ACI without comparing other localizations, proving no clinical worsening over the time in patellar lesion, despite his previous finding at mid-term results [36]. Ebert et al. showed a significant difference between femorotibial and patellofemoral KOOS scores [44]. In a recent study, Niemeyer et al. presented no significantly better results for patellar defects than femoral using the matrix-associated ACI with spheroid technology [20].

With the increasing number of ACI procedures, there are now many studies focusing attention on the size of the defect. In all of the available studies, the focus of interest is the absolute defect size measured intra-operatively. According to actual recommendations, defect size from 2.5 to 3 cm^2^ and larger provides a potential indication for ACI. Previously, it was thought that treatment of large defects was associated with an increased risk of failure [24]. However, there are now many studies that did not find any correlation between outcomes and the absolute defect size [14, 23, 27]. In our study, we also did not find any correlation between outcome and absolute defect size.

To our knowledge, there is no study investigating the relative defect size as the share of the defect on the whole cartilage layer of the knee compartment. Therefore, in our study, we analyzed the absolute and relative defect size, which we obtained by comparing the defect size measured in the MRI in relation to the whole size of the knee compartment. We showed that the same absolute defect size at medial femoral condyle and patella has a significantly different share on the total size of the joint compartment (relative defect size) (*p* < 0.05). Nevertheless, it had not been proven in our further analysis if the relative defect size had no significant influence on the clinical outcome (*p* > 0.05). According to our results, neither the absolute nor the relative defect size has a significant influence on outcomes and could not explain the inferiority of the patellar clinical outcome.

A potential negative effect of the worse clinical outcome in patellar defects is the complexity of the patellofemoral joint. Maltracking or patella instability is causing most of the patellar cartilage defects. Concomitant surgery is often needed. In this study, we could not find negative effects of concomitant surgery in general in both groups. Also, there was no negative effect regarding the treatment of patella instability. In 8 patellar patients, additional stabilization of the patella was performed without worse clinical outcomes (*p* > 0.005).

A limitation of our study is the relatively small number of patients (*n* = 50), with 25 in each group and a relatively short follow-up of three years. A larger study would be helpful to have stronger reliability in our findings and would enable the possibility of getting the significant results for relative defect sizes. Long-term follow-up would also bring us more information about the durability and further development of our results. The next limitation could be the missing matching for aetiology of the defects. When interpreting the results of the present study, lack of randomization should be kept in mind, which reduces the level of evidence from I to III.

## Conclusion

Third-generation ACI provides clinical benefits for both patellar and femoral defects. In a follow-up of three years, ACI-treated femoral cartilage defects showed superior clinical outcomes compared with patellar defects. No significant influence of the relative or absolute defect size was found in either femoral or patellar cartilage defects.
